# The Associations between Infertility and Antioxidants, Proinflammatory Cytokines, and Chemokines

**DOI:** 10.1155/2018/8354747

**Published:** 2018-07-16

**Authors:** Dorota Chyra-Jach, Zbigniew Kaletka, Michał Dobrakowski, Anna Machoń-Grecka, Sławomir Kasperczyk, Ewa Birkner, Aleksandra Kasperczyk

**Affiliations:** ^1^Department of Biochemistry, School of Medicine with the Division of Dentistry in Zabrze, Medical University of Silesia, ul. Jordana 19, 41-808 Zabrze, Poland; ^2^Department and Clinic of Urology, School of Medicine with the Division of Dentistry in Zabrze, Medical University of Silesia, ul. 3 Maja 13-15, 41-800 Zabrze, Poland

## Abstract

The aim of the study was to evaluate the parameters of oxidative stress and antioxidant defense in relation to the levels of proinflammatory cytokines and chemokines in patients diagnosed with oligozoospermia and asthenozoospermia. Based on the basic parameters of the spermogram, the examined group (*n* = 243) was divided into three groups: oligospermic group (sperm count less than 15 × 10^6^/ml) consisting of 152 men, astenozoospermic group (less than 40% of progressively moving sperm cells) consisting of 142 men, and oligoastenozoospermic group (both criteria met) consisting of 90 men. The control group consisted of 103 males with normal semen profile according to the WHO criteria. Total superoxide dismutase (SOD) activity in seminal plasma and spermatozoa lysate was significantly lower by 12% and 22%, respectively, in males with oligospermia than in the control group. Analogically, Mn-SOD activity in spermatozoa lysate was significantly lower in males with oligospermia, asthenospermia, and oligoasthenospermia by 44%, 32%, and 45%, respectively. By contrast, CuZn-SOD activity in spermatozoa lysate was significantly higher in males with oligospermia by 60%. The activity of glutathione peroxidase (GPx) in seminal plasma was also significantly higher in males with oligospermia and oligoasthenospermia by 56% and 78%, respectively. The level of malondialdehyde (MDA) in seminal plasma was significantly higher in males with asthenospermia than in the control group by 12%. By contrast, the level of MDA in spermatozoa lysate was significantly lower in males with oligospermia, asthenospermia, and oligoasthenospermia by 26%, 20%, and 26%, respectively. The level of interleukin- (IL-) 8 in seminal plasma was significantly higher in males with asthenospermia and oligoasthenospermia by 64% and 67%, respectively. Abnormalities in spermogram, such as oligospermia, asthenospermia, and oligoasthenospermia, may be related to a decreased activity of Mn-SOD in spermatozoa and increased levels of chemokines in seminal plasma.

## 1. Introduction

The failure to conceive after one year of regular, unprotected intercourse with the same partner is defined as infertility [1]. The male factor is the cause of infertility in couples in approximately 30–40% of cases [2]. Defective sperm function is the most common cause of male infertility. Abnormal semen parameters include decreased sperm concentration, impaired motility, and altered morphology. There are many possible endogenous and exogenous factors that influence sperm quality. Many studies indicate that oxidative stress should be regarded as a plausible cause of idiopathic male infertility [3].

In spermatozoa, the NADPH oxidase at the level of the sperm plasma membrane and the NADH-dependent oxidoreductase at the mitochondrial level are the two major sources of reactive oxygen species (ROS). In seminal plasma, the main exogenous sources of ROS are radiation and toxins, including tobacco smoke and alcohol, while the main endogenous sources of ROS include the pathophysiologic effects of varicocele, accumulation of damaged spermatozoa with excess residual cytoplasm, and immune cells. Various intracellular or extracellular stimuli, such as infection or inflammation, may recruit and activate peroxidase-positive leukocytes, including polymorphonuclear leukocytes and macrophages that originate from the prostate and seminal vesicles. These cells are able to discharge up to 100 times more ROS than normal as a result of a respiratory burst. Consistently, many studies indicate a correlation between decreased sperm function and elevated levels of proinflammatory cytokines [4].

At low concentrations, ROS play an important role in capacitation, hyperactivation, acrosome reaction, and spermatozoa-oocyte fusion. However, elevated levels of ROS override antioxidant defenses and lead to a damage to biomolecules such as lipids, proteins, and nucleic acids [5]. Human spermatozoa are extremely vulnerable to oxidative attack because they contain high amounts of polyunsaturated fatty acids and little cytoplasm sequestering antioxidants. Therefore, human seminal plasma serves as a source of antioxidants. In this microenvironment, antioxidant enzymes, such as superoxide dismutase (SOD) and catalase (CAT), can be found. Besides, seminal plasma contains high levels of nonenzymatic antioxidants, such as ascorbate or thiol groups [6].

Oxidative stress-induced loss of membrane integrity, increased cell permeability, enzyme inactivation, structural damage of DNA, and cell death may be associated with decreased sperm count and motility [1, 4]. In light of this, we decided to evaluate the parameters of oxidative stress and antioxidant defense in relation to the levels of proinflammatory cytokines and chemokines in males diagnosed with oligospermia and asthenospermia.

## 2. Materials

The study group consisted of 346 males living in Upper Silesia (Poland) who had attended an andrology clinic to diagnose infertility. Information about the fertile abilities of men was provided by the spermogram test. The seminal samples were collected by masturbation after 3 days of abstinence on the same day in the morning before the first meal. All of the semen specimens were analyzed according to WHO standards [7], including the assessment of seminal volume, sperm cell density, total sperm cell count, motility, supravital eosin staining (for percentage of live spermatozoa), and number of peroxidase-positive cells. Additively, we analyzed percentages of motile spermatozoa after 24 hours and progressive motile spermatozoa after 24 hours.

Based on the basic parameters of the spermogram, the examined group was selected (*n* = 243). Subjects in that group were divided into three groups: oligospermic group (sperm count less than 15 × 10^6^/ml) consisting of 152 men, astenozoospermic group (less than 40% of progressively moving sperm cells) consisting of 142 men, and oligoastenozoospermic group (both criteria met) consisting of 90 men. The control group consisted of 103 males with normal semen profile according to the WHO criteria [7]. The exclusion criteria were defined as follows: drug consumption (including antioxidant medications), smoking habits, alcohol abuse, and a history of any chronic disease, such as diabetes, coronary artery disease, or malignant neoplasm.

The experimental set-up has been approved by the Bioethics Committee of the Medical University of Silesia in Katowice (KNW/0022/KB1/I/13/09).

## 3. Methods

### 3.1. Sample Preparation and Semen Analysis

The semen specimens were analyzed according to WHO standards [7].

After complete liquefaction, seminal plasma was separated from the spermatozoa by centrifugation at 6000*g* for 10 minutes. The supernatants obtained were stored at −75°C until required for the biochemical analysis. In addition, a 10% spermatozoa lysate in bidistilled water was made.

### 3.2. Biochemical Analysis

#### 3.2.1. Antioxidant Enzymes

The method of Oyanagui [8] was used to measure the activities of SOD, CuZn-SOD, and Mn-SOD in seminal plasma and spermatozoa. The enzymatic activity of SOD was expressed in nitric units. The activity of SOD is equal to 1 nitric unit (NU) when it inhibits nitric ion production by 50%. Activities of SOD and isoenzymes, Mn-SOD and CuZn-SOD, in seminal plasma were expressed in NU/ml and in NU/dl packed spermatozoa. The seminal plasma glutathione peroxidase (GPx) activity was measured by the kinetic method of Paglia and Valentine [9]. The GPx activity was expressed in U/l. The activity of seminal plasma glutathione-S-transferase (GST) was measured according to the kinetic method of Habig and Jakoby [10]. The GST activity was expressed as moles of thioether produced per minute per liter of seminal plasma (U/l). The activities of glucose-6-phosphate dehydrogenase (G6PD) and glutathione reductase (GR) in seminal plasma were measured according to Richterich [11]. G6PD and GR activity was expressed as *μ*mol of NADPH produced and utilized, respectively, per minute per liter of seminal plasma (U/l). CAT activity in seminal plasma was measured by the method of Johansson and Borg [12]. The activity of catalase was expressed as U/l.

#### 3.2.2. Nonenzymatic Antioxidants

Total antioxidant capacity (TAC) was measured according to Erel [13]. Data were shown as mmol/l. The levels of uric acid (UA), bilirubin, and albumin were determined in seminal plasma by colorimetric methods. For uric acid and bilirubin, concentrations are provided in mg/dl, and albumin is expressed in g/ml. The concentration of thiol groups (SH) in seminal plasma was determined by Koster et al. [14]. The results were shown as *μ*mol/l.

#### 3.2.3. Markers of Oxidative Stress

The level of malondialdehyde (MDA) in seminal plasma and spermatozoa lysate according to Ohkawa et al. [15]. TBARS values are expressed as malondialdehyde (MDA) equivalents. Concentrations were given in *μ*mol/l in seminal plasma and *μ*mol/dl in packed spermatozoa. The lipofuscin (LPS) concentration was determined in seminal plasma according to Jain [16]. Values were presented as relative units (relative fluorescence lipid extract, RF). Total oxidant status (TOS) was measured in seminal plasma according to Erel [17]. Data were shown in *μ*mol/l.

#### 3.2.4. Cytokines

The levels of IL-1*β*, IL-6, IL-8, IL-12, TNF-*α*, MCP-1, and MIP-1*β* were measured in seminal plasma using a Bio-Plex 200 System (Bio-Rad Laboratories Inc., USA) according to the manufacturer's instructions. Data were presented in pg/ml.

### 3.3. Statistical Analysis

A database was created in MS Excel 2007. Statistical analysis was performed using Statistica 10.0 PL software. Statistical methods included mean and standard deviation (SD) for normal distribution and median and interquartile range (IQR) for abnormal distribution. Shapiro-Wilk's test was used to verify normality and Levene's test to verify homogeneity of variances. Statistical comparisons between groups were made by a *t*-test, *t*-test with a separate variance, or Mann–Whitney *U* test (nonparametric test). Spearman's coefficient R for nonparametric correlation was calculated. A value of *p* < 0.05 was considered significant.

## 4. Results

Mean age in the control and examined groups did not differ significantly (Table 1). Differences between the control group and examined groups in terms of the semen volume, pH, count, and motility are presented in Table 1. The numbers of peroxidase-positive cells were within the normal ranges (<1 × 10^6^/ml) in all examined groups and did not differ among them.

Total SOD activity in seminal plasma and spermatozoa lysate was significantly lower by 12% and 22%, respectively, in males with oligospermia than in the control group. Analogically, Mn-SOD activity in spermatozoa lysate was significantly lower in males with oligospermia, asthenospermia, and oligoasthenospermia by 44%, 32%, and 45%, respectively. By contrast, CuZn-SOD activity in spermatozoa lysate was significantly higher in males with oligospermia by 60%. The activity of GPx in seminal plasma was also significantly higher in males with oligospermia and oligoasthenospermia by 56% and 78%, respectively (Table 2).

The level of UA in seminal plasma was significantly higher in males with oligoasthenospermia than in the control group by 19%, while the level of albumin in seminal plasma was significantly lower in males with oligospermia by 12% (Table 3).

The level of MDA in seminal plasma was significantly higher in males with asthenospermia than in the control group by 12%. By contrast, the level of MDA in spermatozoa lysate was significantly lower in males with oligospermia, asthenospermia, and oligoasthenospermia by 26%, 20%, and 26%, respectively (Table 4).

The level of IL-12 in seminal plasma was significantly lower in males with oligoasthenospermia than in the control group by 50%. At the same time, the level of IL-8 in seminal plasma was significantly higher in males with asthenospermia and oligoasthenospermia by 64% and 67%, respectively. Analogically, the level of MCP-1 in seminal plasma was significantly higher by 47% and 64%, respectively. The level of MIP-1*β* in seminal plasma was significantly higher in males with oligospermia, asthenospermia, and oligoasthenospermia than in the control group by 57%, 47%, and 49%, respectively (Table 5).

Spearman correlations showed positive correlations between the sperm cell count and motility and spermatozoa Mn-SOD activity (*R* = 0.26–0.51, *p* < 0.05) and spermatozoa MDA (*R* = 0.23–0.44, *p* < 0.05) as well as albumin concentration in seminal plasma (*R* = 0.15–0.25, *p* < 0.05). By contrast, the level of MDA, MCP-1, and IL-8 in seminal plasma negatively correlated with motility (R between −0.11 and −0.21, *p* < 0.05) (Table 6).

## 5. Discussion

The activity of the antioxidant defense system in spermatozoa is limited by the low amount of their cytoplasm [18]. Nevertheless, human seminal plasma is considered as an important source of antioxidants. SOD is believed to be the first enzymatic line of antioxidant defense [1]. SOD prevents lipid peroxidation of plasma membrane through superoxide anion utilization by converting it into hydrogen peroxide. In order to prevent the toxic action of hydrogen peroxide, SOD should be conjugated with CAT or GPx [18]. Utilization of hydrogen peroxide by GPx depletes GSH pool, a cofactor which is converted to oxidized glutathione (GSSG). The recycling of GSSG into its reduced form depends on the activity of GR which needs NADPH as a reducing cofactor. The main source of NADPH is the pentose phosphate cycle in which G6PD transforms glucose-6-phosphate into phospho-6-gluconolactone releasing NADPH [19].

It has been established that SOD plays a major role in maintaining sperm viability and its activity in spermatozoa is positively correlated with duration of sperm motility [2]. Consistently, we showed positive correlations between SOD activity in spermatozoa lysate and sperm volume, sperm cell count, rapid progressive motility after 1 hour, and motile spermatozoa after 24 hours. At the same time, the percentages of nonlinear progressive and unprogressive motile spermatozoa after 1 hour correlated negatively with SOD activity in spermatozoa lysate. We reported also lower activities of SOD in seminal plasma and spermatozoa lysate of males with oligospermia than in the controls. These results confirm the proposed protective role of SOD against oxidative stress in semen. In this context, the activity of manganese isoenzyme of SOD seems to be crucial for sperm quality maintenance because we reported lower activities of Mn-SOD in spermatozoa lysate of males with oligospermia, asthenospermia, and oligoasthenospermia than in the control group. Additively, the activity of Mn-SOD positively correlated with sperm volume, sperm cell count, and sperm motility in the examined population. Consistently, in our previous study, we showed a negative association between SOD activities, including Mn-SOD, in spermatozoa and oxidative stress measured as a TOS level [20]. Mn-SOD is localized in the mitochondrial matrix. Mitochondria are responsible for energy production via the oxidative phosphorylation pathway and are one of the major sources of chronic ROS production under physiological conditions and compromised by severe and prolonged oxidative stress. Decreased Mn-SOD activity promotes generation of oxidants which inactivate enzymes and damage mtDNA leading to the disruption of mitochondrial integrity [21]. Ultimately, mitochondrial dysfunction may lead to ATP pool depletion and sperm motility impairment. Mitochondrial dysfunction associated with decreased oxidative metabolism may be an explanation for observed simultaneously paradoxically lower MDA level in spermatozoa lysate of males with oligospermia, asthenospermia, and oligoasthenospermia than in the control group. The second explanation for lower MDA level in spermatozoa is its possible leakage form damaged sperm cells due to oxidative stress and peroxidation of cell membrane lipids. Additively, in males with oligospermia, lower MDA level may be also due to a higher activity of CuZn-SOD in spermatozoa lysate. This SOD isoenzyme is localized in the cytosol with a smaller fraction in the intermembrane space of mitochondria [21]. Elevation of its activity might be a result of a compensatory defense mechanism. Higher activities of GPx in seminal plasma of males with oligospermia and oligoasthenospermia than in the control group should be interpreted in the same way. At the same time, in males with asthenospermia, antioxidant defense in seminal plasma seems to be insufficient because an elevated level of MDA in that group was observed. The levels and activities of remaining parameters of oxidative stress and antioxidant enzymes did not differ between the control and examined groups.

Results of other studies on this topic are only partially in concordance with our observations. Marzec-Wróblewska et al. [2] reported lower SOD activity in males with pathological spermogram than in the normozoospermic males. SOD activity was also negatively associated with semen volume and positively associated with rapid progressive motility, nonprogressive motility, and sperm concentration. Similarly, in a study of Zelen et al. [5], the activities of SOD and CAT were significantly lower in the seminal plasma of the oligozoospermic, astenozoospermic, and teratozoospermic patients compared to the fertile controls, while the level of MDA was higher in the infertile subjects. Analogical results were shown in a study of Shiva et al. [18] who reported a significant increase in the MDA levels in asthenozoospermics and teratozoospermics as compared to progressively motile and morphologically normal groups, respectively. A positive correlation between SOD activity and sperm count and total progressive motility was also found. Authors concluded that decline in SOD activity might be involved in the abnormal semen quality. On the other hand, Abdallah et al. [6] reported elevated activity of SOD in azoospermic, oligoasthenozoospermic, and asthenozoospermic males compared to normozoospermic ones postulating that SOD expression is upregulated in response to defective spermatogenesis or hormonal deficiency. In other analogous studies, unchanged, elevated, and decreased activities of SOD, CAT, and GPx were found in semen of infertile males compared to the fertile controls [6]. The discrepancies between studies may be due to the different study protocols and a result of action of many factors influencing antioxidant enzyme expression and activities. In light of this, as proposed Tavilani et al. [22], lack of protection against lipid peroxidation in semen of infertile males may be not due to the alteration in the activity of a particular antioxidant enzyme but rather due to a noncoordination between several of them. Consistently, Micheli et al. [23] suggested that the alteration of a single parameter of oxidative stress/antioxidant system does not have enough clinical value to estimate the male fertilizing potential.

The associations between fertility impairment and seminal nonenzymatic antioxidant defense seem to be as complex as those between antioxidant enzyme activities and spermogram parameters. Seminal plasma and spermatozoa contain nonenzymatic ROS scavengers, such vitamins, glutathione, uric acid, and albumin [24]. The antioxidant properties of albumin are attributed to the thiol groups of its cysteine residues. Albumin is believed also to sequester prooxidant molecules and redox-active metals [25]. Consistently, bovine serum albumin has been shown to protect membrane integrity of sperm cells from heat shock during freezing thawing of canine semen [26]. In light of this, significantly lower seminal plasma albumin level in males with oligospermia than in the controls should be interpret as attenuation of antioxidant defense. The antioxidant properties of uric acid are less unequivocal. On the one hand, uric acid acts as a scavenger of ROS being regarded as a main antioxidant in human plasma. On the other hand, uric acid may be a prooxidant under conditions of oxidative stress [27]. Lahnsteiner et al. [28] reported that uric acid is the primary antioxidant in semen of brown trout. Therefore, higher levels of uric acid in males diagnosed with oligoasthenospermia than in the controls may be interpret as an effect of compensatory defense mechanism; however, elevation of this metabolite level may be also due to increased purine degradation.

The inflammatory process within the male genitourinary tract was found to reduce fertilizing potential of mature spermatozoa. Recruitment of immune cells to the site of inflammation results in the release of reactive oxygen intermediates and proinflammatory cytokines by activated neutrophils and macrophages [29]. Consistently, we reported higher levels of IL-8, MCP-1, and MIP-1*β* in males with asthenospermia, oligoasthenospermia, and oligospermia than in the control group. All of these compounds play a role in chemokines [30]. On the other hand, the levels of proinflammatory cytokines were not simultaneously higher in males with abnormal spermogram. Camejo et al. [31] reported higher IL-6 concentration in seminal plasma of infertile men compared to fertile men, while the level of TNF-*α* did not differ between studied groups. At the same time, there was a positive correlation between the levels of IL-6 in seminal plasma and the levels of lipid peroxidation of the sperm membranes. However, TNF-*α* and IL-6 concentrations did not correlate with sperm parameters, such as normal morphology, sperm concentration, and motility, in that study. Consistently, Frączek et al. [29] concluded that proinflammatory cytokines per se are unable to cause oxidative stress in semen to the level of membrane oxidative damage. The discrepancies between studies are probably due to the complex dependences between cytokines which act inhibitory or synergistic in an array [32].

## 6. Conclusions

Abnormalities in spermogram, such as oligospermia, asthenospermia, and oligoasthenospermia, may be related to decreased activity of Mn-SOD in spermatozoa and increased levels of chemokines in seminal plasma.

## Figures and Tables

**Table 1:** Age, semen analysis in control and study groups; *p* value—*t*-test.

	Control *n* = 107		Oligospermia *n* = 155				Asthenospermia *n* = 146				Oligoasthenospermia *n* = 91			
	Mean	SD	Mean	SD	Relative change	*p* value	Mean	SD	Relative change	*p* value	Mean	SD	Relative change	*p* value
Age (years)	33	6	34	5	2%	0.432	34	6	3%	0.256	34	5	2%	0.403
Sperm volume (ml)	3.70	1.73	3.58	1.53	−3%	0.541	3.42	1.63	−8%	0.186	3.41	1.58	−8%	0.214
pH value	7.56	0.08	7.57	0.09	0%	0.646	7.57	0.08	0%	0.783	7.56	0.09	0%	0.915
Sperm cells count in 1 ml (mln/ml)	79.5	60.1	4.39	4.51	**−94%**	**<0.001**	25.4	42.8	**−68%**	**<0.001**	3.81	4.68	**−95%**	**<0.001**
Total sperm cells count (mln)	269	189	15.5	19.9	**−94%**	**<0.001**	73.9	122	**−73%**	**<0.001**	12.5	19.8	**−95%**	**<0.001**
Total motility after 1 hour (% motile)	58.1	9.48	32.4	20.6	**−44%**	**<0.001**	22.9	14.0	**−61%**	**<0.001**	18.3	14.1	**−69%**	**<0.001**
Rapid progressive motility (a) after 1 hour (%)	26.0	9.96	9.16	7.82	**−65%**	**<0.001**	6.75	5.97	**−74%**	**<0.001**	5.14	5.55	**−80%**	**<0.001**
Slow progressive motility (b) after 1 hour (%)	18.0	6.81	9.53	8.11	**−47%**	**<0.001**	6.76	7.13	**−62%**	**<0.001**	4.77	4.98	**−73%**	**<0.001**
Progressive motility (a + b) after 1 hour (%)	44.0	9.74	18.7	14.22	**−57%**	**<0.001**	13.5	10.66	**−69%**	**<0.001**	9.9	9.41	**−77%**	**<0.001**

*p* < 0.001.

**Table 2:** Antioxidant enzyme activity in seminal plasma and spermatozoa (SOD: superoxide dismutase; CAT: catalase; GR: glutathione reductase; GPx: glutathione peroxidase; GST: glutathione-S-transferase; G6PD: glucose-6-phosphate dehydrogenase; *p* value—*t*-test.

	Control		Oligospermia				Asthenospermia				Oligoasthenospermia			
	Mean	SD	Mean	SD	Relative change	*p* value	Mean	SD	Relative change	*p* value	Mean	SD	Relative change	*p* value
Total SOD activity (NU/ml)	183	55.5	162	55.6	**−12%**	**0.004**	169	53.3	−8%	0.062	170	50.8	−7%	0.087
Mn-SOD activity (NU/ml)	37.6	44.0	31.7	36.1	−16%	0.317	37.1	43.6	−1%	0.935	32.9	34.2	−12%	0.504
CuZn SOD activity (NU/ml)	143	49.8	129	52.2	−10%	0.063	133	52.0	−7%	0.210	138	43.6	−3%	0.555
CAT activity (U/l)	607	460	573	336	−6%	0.547	608	413	0%	0.986	579	352	−5%	0.685
GR activity (U/l)	78.8	85.7	70.0	71.2	−11%	0.378	69.0	73.3	−12%	0.341	70.3	74.9	−11%	0.474
GPx activity (U/l))	118	210	184	276	**56%**	**0.039**	187	301	58%	0.051	210	284	**78%**	**0.007**
GST activity (U/l)	3.75	2.96	3.34	2.05	−11%	0.837	3.29	2.18	−12%	0.187	3.64	2.30	−3%	0.787
G6PD activity (U/l)	64.8	53.0	63.7	45.9	−2%	0.701	55.2	44.9	−15%	0.251	58.8	46.0	−9%	0.531
Total SOD activity (NU/dl packed spermatozoa)	152	75.4	119	75.3	**−22%**	**0.045**	128	78.8	−16%	0.198	118	75.8	−22%	0.106
Mn-SOD activity (NU/dl packed spermatozoa)	135	66.7	75.3	51.5	**−44%**	**<0.001**	90.9	57.7	**−32%**	**0.007**	73.9	51.8	**−45%**	**0.001**
CuZn SOD activity (NU/dl packed spermatozoa)	33.7	19.4	53.9	42.0	**60%**	**0.019**	46.2	35.9	37%	0.095	48.6	41.6	44%	0.093

*p* < 0.05.

**Table 3:** Antioxidant reserves in seminal plasma and spermatozoa (thiol group concentration (SH concentration), total antioxidant capacity (TAC); *p* value—*t*-test.

	Control		Oligospermia				Asthenospermia				Oligoasthenospermia			
	Mean	SD	Mean	SD	Relative change	*p* value	Mean	SD	Relative change	*p* value	Mean	SD	Relative change	*p* value
SH concentration (*μ*mol/l)	211	86.6	198	84.6	−6%	0.304	196	91.6	−7%	0.279	199	87.7	−6%	0.432
TAC (mmol/l)	1.33	0.24	1.31	0.30	−1%	0.785	1.34	0.28	1%	0.814	1.35	0.33	2%	0.690
Uric acid concentration (mg/dl)	4.39	1.40	4.96	2.35	13%	0.079	4.90	2.54	12%	0.142	5.20	2.89	**19%**	**0.045**
Bilirubin (mg/dl)	0.12	0.17	0.10	0.13	−15%	0.445	0.14	0.23	17%	0.567	0.12	0.16	3%	0.906
Albumin (g/ml)	0.49	0.16	0.43	0.15	**−12%**	**0.007**	0.45	0.16	−8%	0.052	0.44	0.17	−10%	0.118

*p* < 0.05.

**Table 4:** Parameters related to oxidative stress intensity in seminal plasma and spermatozoa (MDA: malondialdehyde; TOS: total oxidant status); *p* value—*t*-test.

	Control		Oligospermia				Asthenospermia				Oligoasthenospermia			
	Mean	SD	Mean	SD	Relative change	*p* value	Mean	SD	Relative change	*p* value	Mean	SD	Relative change	*p* value
MDA concentration (*μ*mol/dl packed spermatozoa)	0.56	0.19	0.41	0.09	**−26%**	**<0.001**	0.45	0.12	**−20%**	**0.006**	0.42	0.09	**−26%**	**0.002**
MDA concentration (*μ*mol//l)	2.41	0.98	2.63	1.18	9%	0.125	2.71	1.13	**12%**	**0.031**	2.69	1.20	11%	0.087
Lipofuscin (RF)	3.92	1.57	3.84	1.35	−2%	0.705	3.86	1.26	−1%	0.786	3.91	1.33	0%	0.971
TOS (*μ*mol/l)	11.0	13.8	8.23	13.2	−25%	0.248	9.72	13.5	−12%	0.591	8.62	14.8	−22%	0.409

*p* < 0.05.

**Table 5:** Concentrations of cytokines in seminal plasma (IL-1*β*: interleukin 1*β*; IL-6: interleukin 6; IL-8: interleukin 8; IL-12 interleukin 12; MCP-1: monocyte chemoattractant protein-1; MIP-1*β*: macrophage inflammatory protein 1-*β*; TNF-*α*: tumor necrosis factor *α*; *p*-value—Mann–Whitney *U* test.

	Control		Oligospermia				Asthenospermia				Oligoasthenospermia			
	Median	IQR	Median	IQR	relative change	*p* value	Median	IQR	relative change	*p* value	Median	IQR	relative change	*p* value
IL-1*β* (pg/ml)	1.21	2.61	1.98	10.52	64%	0.164	1.66	9.90	37%	0.134	1.65	9.93	36%	0.198
IL-6 (pg/ml)	9.97	13.0	13.5	18.7	35%	0.161	15.90	16.4	59%	0.177	17.0	17.4	71%	0.064
IL-8 (pg/ml)	155	157	242	371	56%	0.092	254	437	**64%**	**0.017**	259	423	**67%**	**0.038**
IL-12 (pg/ml)	2.80	3.72	1.56	3.40	−44%	0.108	2.22	3.74	−21%	0.296	1.39	2.99	**−50%**	**0.041**
MCP-1 (pg/ml)	992	1181	1347	1411	36%	0.221	1457	1387	**47%**	**0.038**	1624	1467	**64%**	**0.041**
MIP-1*β* (pg/ml)	50.9	31.0	79.8	111	**57%**	**0.006**	74.9	76.7	**47%**	**0.013**	75.8	72.2	**49%**	**0.016**
TNF-*α* (pg/ml)	4.13	3.39	3.73	4.16	−10%	0.326	3.88	3.68	−6%	0.361	3.67	4.16	−11%	0.318

IQR: interquartile range, *p* < 0.05.

**Table 6:** Spearman correlations between semen parameters and oxidative stress and cytokines.

	Seminal plasma								Spermatozoa			
	Total SOD	Mn-SOD	GPx	MDA	Albumin	IL-8	IL-12 (p70)	MCP-1	Total SOD	Mn-SOD	CuZn-SOD	MDA
Sperm cell count in 1 ml	0.17	0.15			0.22		0.19		0.31	0.51		0.44
Total sperm cell count	0.12	0.15			0.21		0.18		0.33	0.51		0.44
Total motility after 1 hour				−0.14		−0.18		−0.21		0.26		0.23
Rapid progressive motility (a) after 1 hour								−0.24	0.25	0.47		0.36
Slow progressive motility (b) after 1 hour				−0.21	0.20		0.20	−0.18			−0.27	
Progressive motility (a + b) after 1 hour				−0.11	0.15			−0.22		0.31	−0.24	0.34
Motile spermatozoa after 24 hours			−0.18		0.29				0.30	0.44		0.26
Progressive motility after 24 hours			−0.16	−0.11	0.25					0.39		0.31

*R*, *p* < 0.05.

## Data Availability

The data used to support the findings of this study are available from the corresponding author upon request.
